# Both task-irrelevant and task-relevant information trigger reactive conflict adaptation in the item-specific proportion-congruent paradigm

**DOI:** 10.3758/s13423-022-02138-5

**Published:** 2022-06-29

**Authors:** Giacomo Spinelli, J. Bruce Morton, Stephen J. Lupker

**Affiliations:** 1 Dipartimento di Psicologia, Università degli Studi di Milano-Bicocca, Piazza dell’Ateneo Nuovo 1, 20126 Milan, Italy; 2 Department of Psychology, University of Western Ontario, London, Ontario Canada

**Keywords:** Conflict adaptation, Reactive control, Item-specific proportion-congruent effect, Stroop

## Abstract

Adapting attention flexibly is a fundamental ability of the human control system. In the color-word Stroop task, for example, congruency effects are typically smaller for colors and words that appear mainly in incongruent stimuli (mostly-incongruent items) than for colors and words that appear mainly in congruent stimuli (mostly-congruent items). At least part of this item-specific proportion-congruent (ISPC) effect is due to a process of reactive conflict adaptation that affords higher selectivity (i.e., more efficient selection of task-relevant information) when a specific stimulus is presented that is frequently associated in the experiment with conflicting task-irrelevant information. What is unclear, however, is whether, normally, this stimulus-specific adaptation is triggered by the task-relevant component, the task-irrelevant component, or both components of the stimulus. In two experiments, using modified color-word (Experiment 1) and spatial (Experiment 2) Stroop tasks that allowed task-relevant and task-irrelevant triggering processes to be dissociated, we found that the two processes have approximately equivalent impacts. Because these results were obtained in experiments imposing no limitations on the processes potentially contributing to the ISPC effect, these results challenge claims that the ISPC effect involves conflict-adaptation processes only in special situations. The ISPC effect may involve conflict-adaptation processes in most situations, with both task-relevant and task-irrelevant information triggering such processes.

**Supplementary Information:**

The online version contains supplementary material available at 10.3758/s13423-022-02138-5.

## Introduction

Adjusting performance based on the characteristics of the stimuli encountered in a particular situation is a remarkable human ability (Chiu & Egner, 2019). One paradigm for examining this type of ability is the Item-Specific Proportion-Congruent (ISPC) paradigm (Jacoby et al., 2003). In this paradigm, typically implemented in color-word and picture-word versions of the Stroop (1935) task, congruency effects (i.e., the response-time (RT) difference between incongruent/conflicting stimuli, e.g., the word RED presented in blue, and congruent/non-conflicting stimuli, e.g., the word RED presented in red) are larger for mostly-congruent (MC) items (e.g., when RED is presented most often in red in that specific task), items for which conflict is infrequent, than for mostly-incongruent (MI) items (e.g., when GREEN is presented most often in yellow), items for which conflict is frequent.

The ISPC effect is often interpreted as reflecting different processes depending on the version of the ISPC paradigm that produced it. For example, Chiu et al. (2017) compared two versions of the ISPC paradigm using a face-name Stroop task. In one version, the “stimulus-response learning condition,” it was possible to produce an ISPC effect by adjusting control appropriately based on the frequency of conflict associated with either the presented face and/or name (the task-relevant and task-irrelevant stimulus components in that task, respectively), a reactive conflict-adaptation process. It was also possible to produce an ISPC effect by learning and using associations between a particular name (e.g., CLOONEY) and the response typically made to stimuli containing that name (e.g., “Pitt”), a contingency-learning process (Schmidt & Besner, 2008). Chiu et al. (2017) ultimately concluded that the latter process produced the (relatively large) ISPC effect obtained in that version.

In another version of the ISPC paradigm that Chiu et al. examined, the “stimulus-control learning condition,” an ISPC effect could only be produced by a reactive conflict-adaptation process based on the frequency of conflict associated with the presented face (the task-relevant component). That is, contingency learning could not produce the effect in this situation because contingencies between task-relevant components and responses are fixed (e.g., a Pitt face requires a “Pitt” response in all circumstances; but for a counterargument, see Schmidt, 2019). A smaller ISPC effect with a different neural profile was obtained, presumably based on a reactive conflict-adaptation process triggered by the task-relevant stimulus component (see also Bugg, 2015; Bugg et al., 2011; Bugg & Hutchison, 2013).

Although these results suggest a role for reactive conflict adaptation in the ISPC paradigm, they restrict this role to versions of the paradigm that prevent contingency learning from contributing to the ISPC effect. Further, in those versions, the conflict-adaptation process examined appears to be triggered by the task-relevant components, as the task-irrelevant components only provided weak cues to conflict frequency (for an exception, see Bugg & Hutchison, 2013, Experiment 3). However, in Jacoby et al.’s (2003) original paradigm, not only could contingency learning have contributed to the ISPC effect, but, more relevantly, task-irrelevant and task-relevant components provided equally strong cues to conflict frequency (e.g., both the MI word GREEN and the MI color yellow were associated with frequent conflict). Therefore, either component (or both) could be potential conflict-adaptation trigger(s) (e.g., recognizing either an MI *word* or an MI *color* would induce higher selectivity). Note also that Spinelli and Lupker (2020) recently demonstrated that even in that version of the ISPC paradigm, conflict adaptation does play a role, although their design did not permit a determination of which component was a trigger.

The goal of the present experiments was to examine these issues more fully. Specifically, we intended to examine how conflict-adaptation triggers are used when contingency learning is a viable option in the task, but not for the crucial stimuli (the “transfer set,” as described below), and when use of neither trigger is favored, as in the original ISPC paradigm. Because that ISPC paradigm does not impose limitations on the processes potentially contributing to the ISPC effect, that version is considered a fundamental tool for establishing how those processes are used normally (Bugg, 2015). In order to address these issues, it is important, first, to understand how the ISPC effect is explained within a conflict-adaptation framework.

Within that framework, the larger congruency effect for MC items reflects low selectivity, that is, a transient state of the control system associated with reduced efficiency in the selection of task-relevant information induced by recognition that the item’s task-irrelevant and/or task-relevant component is infrequently associated with conflict (e.g., in the color-word Stroop task, the color red, an MC color). Conversely, the relatively smaller congruency effect for MI items reflects a state in which selection of task-relevant information is more efficient, one induced by recognition that the item contains a task-irrelevant and/or task-relevant component frequently associated with conflict (e.g., the color green, an MI color). The result is the emergence of the standard ISPC pattern.

To achieve our goals, we modified the standard ISPC paradigm so that it was possible to dissociate task-irrelevant and task-relevant triggering of conflict adaptation while maintaining the viability of both potential triggers. This modification consisted of adding another set of stimuli to the MC and MI sets traditionally used in the ISPC paradigm. This third set, called the “transfer” set, included stimuli that, having a 50:50 congruent/incongruent ratio overall, were conflict-frequency neutral. The “transfer” task-relevant stimulus components in this set (e.g., the transfer colors), however, were paired with (incongruent) task-irrelevant components from both the MC set (e.g., MC words) and the MI set (e.g., MI words). Similarly, the “transfer” task-irrelevant components in this set (e.g., the transfer words) were paired with (incongruent) task-relevant components from both the MC set (e.g., MC colors) and the MI set (e.g., MI colors). Thus designed, the transfer sets allowed independent examinations of adaptation based on task-irrelevant components (e.g., by comparing MC and MI words appearing with transfer colors) and task-relevant components (e.g., by comparing MC and MI colors appearing with transfer words). Although contingency learning could potentially explain ISPC effects for the standard MC and MI stimuli, contingency learning could not explain any MC-MI differences for items in the transfer set. Finally, in order to establish the generalizability of the results, we extended this modified ISPC paradigm from the traditional, color-word Stroop task (Experiment 1) to a spatial Stroop task (Experiment 2) in which task-relevant and task-irrelevant information had a different nature.

## Experiment 1

### Method

#### Participants

Seventy-four students at the University of Western Ontario participated in the experiment, which was conducted in person, for course credit. Two participants abandoned the experiment before completion, leaving 72 participants (57 females and 15 males; eight left-handed and 64 right-handed; age 17–23 years). After discarding too-fast, too-slow, and incorrect responses (see below), all remaining participants contributed 75% or more of their original observations. Therefore, no other participant was removed from the analyses. These criteria were determined a priori in line with previous work in our laboratory (Spinelli et al., 2020). As revealed by a power analysis performed with G*Power 3.1 (Faul et al., 2009), this sample size essentially has a power of 1 to detect an effect as large as the conflict-adaptation effect reported for a similar manipulation in Bugg and Hutchison’s (2013) Experiment 3 (${\eta}_p^2$ = .21) (see below). All participants were native English speakers and had normal or corrected-to-normal vision. This research was approved by the Research Ethics Board of the University of Western Ontario (protocol # 108956).

#### Materials

Six color names (RED, BLUE, WHITE, GREEN, YELLOW, BLACK) were used as word distractors and the corresponding colors were used as target colors. The frequency of color-word combinations in one of the 12 counterbalancings of the experiment is represented in Table 1 (in the following, this particular counterbalancing will be used for our examples). The stimuli were divided into three sets, with RED, BLUE, and the corresponding colors forming one set, WHITE, GREEN, and the corresponding colors forming another set, and YELLOW, BLACK, and the corresponding colors forming the third set. For each participant, one set (e.g., the red/blue set) served as the MC set, another set (e.g., the white/green set) served as the MI set, and the third set (e.g., the yellow/black set) served as the transfer set.

**Table 1:**
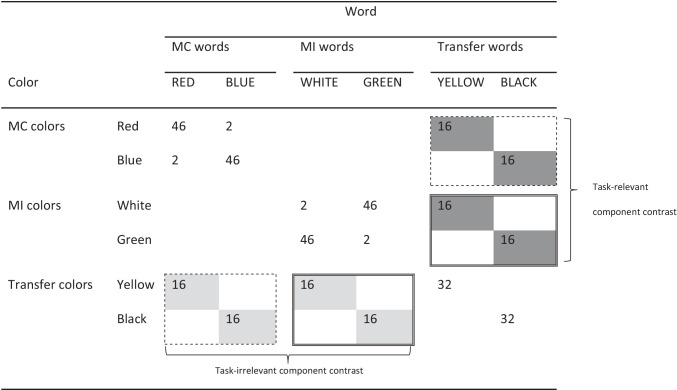
Template for the frequency of color-word combinations in Experiment 1

*Note*. The incongruent items shaded in light grey (i.e., the incongruent items appearing with transfer colors) were used to examine the contrast for the task-irrelevant component triggering of conflict adaptation (i.e., adaptation based on the word). The incongruent items shaded in dark grey (i.e., the incongruent items appearing with transfer words) were used to examine the contrast for the task-relevant component triggering of conflict adaptation (i.e., adaptation based on the color). For each of these contrasts, the items surrounded by a dashed line were used for the MC condition and the items surrounded by a double solid line were used for the MI condition

*MC* mostly-congruent, *MI* mostly-incongruent

In the MC set, each word (e.g., RED) appeared 46 times with its congruent color (e.g., red), twice with the other (incongruent) color in that set (e.g., blue), and 16 times with one of the (incongruent) transfer colors (e.g., yellow). Similarly, in the MC set, each color (e.g., red) appeared 46 times with its congruent word (e.g., RED), twice with the other (incongruent) word in that set (e.g., BLUE), and 16 times with one of the (incongruent) transfer words (e.g., YELLOW). Thus, for each word and for each color in the MC set, the proportion of congruent items was 72%.

Conversely, in the MI set, each word (e.g., WHITE) appeared twice with its congruent color (e.g., white), 46 times with the other (incongruent) color in that set (e.g., green), and 16 times with one of the (incongruent) transfer colors (e.g., yellow). Similarly, in the MI set, each color (e.g., white) appeared twice with its congruent word (e.g., WHITE), 46 times with the other (incongruent) word in that set (e.g., GREEN), and 16 times with one of the (incongruent) transfer words (e.g., YELLOW). Thus, for each word and for each color in the MI set, the proportion of congruent items was 3%.

The main focus, however, concerned the transfer set. In the transfer set, each color (e.g., yellow) appeared 32 times with its congruent word (e.g., YELLOW) as well as 16 times with one of the (incongruent) MC words (e.g., RED) and 16 times with one of the (incongruent) MI words (e.g., WHITE). These incongruent items, shaded in light grey in Table 1, served to examine task-irrelevant triggering of conflict adaptation because they differed in task-irrelevant conflict frequency (low for the transfer colors appearing with MC words, surrounded by a dashed line in Table 1, vs. high for the transfer colors appearing with MI words, surrounded by a double solid line in Table 1). These items, however, did not differ in task-relevant conflict frequency because, for the transfer colors, the proportion of congruent items was fixed at 50%. Further, these items did not differ in contingency learning either, because, although for both MC and MI words a contingency could be learned between the word and a response (the congruent response for MC words, e.g., “red” for the MC word RED, and an incongruent response for MI words, e.g., “green” for the MI word WHITE), the crucial comparison for examining task-irrelevant triggering of conflict adaptation only involved words requiring a different (incongruent) response.

Similarly, in the transfer set, each word (e.g., YELLOW) appeared 32 times with its congruent color (e.g., yellow) as well as 16 times with one of the (incongruent) MC colors (e.g., red) and 16 times with one of the (incongruent) MI colors (e.g., white). These incongruent items, shaded in dark grey in Table 1, served to examine task-relevant triggering of conflict adaptation because they differed in task-relevant conflict frequency (low for the transfer words appearing with MC colors, surrounded by a dashed line in Table 1, vs. high for the transfer words appearing with MI colors, surrounded by a double solid line in Table 1). These items, however, did not differ in task-irrelevant conflict frequency because, for the transfer words, the proportion of congruent items was fixed at 50%. Further, these items did not differ in contingency learning either, because, although for transfer words a contingency could be learned between the word and the congruent response (e.g., for the word YELLOW, “yellow” was a more probable response than either “red” or “white”), the crucial comparison for examining task-irrelevant triggering of conflict adaptation only involved words requiring a different (incongruent) response.

This modification of the ISPC paradigm was inspired by Bugg and Hutchison (2013; see also Bugg et al., 2011), who also used a transfer set to examine task-relevant and task-irrelevant triggering of conflict adaptation independently from one another. Note that, similar to that manipulation, the crucial contrasts in the present manipulation were based on incongruent stimuli, unlike in the original ISPC analysis in which they are based on both congruent and incongruent stimuli. Incongruent stimuli would seem to be the most relevant stimuli for examining conflict-adaptation processes (see, e.g., Bugg et al., 2011). In contrast to Bugg and Hutchison’s manipulation, however, the transfer set in the present manipulation was used throughout the experiment. Doing so permitted an examination of control adjustments as they were being learned (in Bugg and Hutchison’s manipulation, this set only appeared at the end, in a block where the conflict-frequency differences between MC and MI items were attenuated). Further, task-relevant and task-irrelevant triggering of conflict adaptation could be examined simultaneously (in Bugg and Hutchison’s manipulation, the two types of triggers could only be examined in separate experiments; see also Spinelli et al., 2020).

Overall, there were 384 items (160 congruent and 224 incongruent). The proportion of congruent items in the list was thus 41.67%, a little lower than 50%, the typical list-wide congruency proportion in ISPC experiments (e.g., Jacoby et al., 2003). Lists of trials in which incongruent items are more frequent than congruent items induce higher selectivity and, thus, produce somewhat smaller congruency effects (e.g., Logan & Zbrodoff, 1979). However, this state of higher selectivity is assumed to be induced proactively based on the prospective anticipation of forthcoming conflict as opposed to reactively based on the identity of the stimulus components, the type of conflict-adaptation process underlying the ISPC effect (Gonthier et al., 2016; Spinelli & Lupker, 2020). Although proactive and reactive conflict-adaptation processes may interact, they typically co-exist and can be observed independently from one another (Hutchison, 2011; see also Spinelli et al., 2020). Therefore, even assuming that the list-wide congruency proportion of 41.67% of the present experiment was sufficient to induce a proactive conflict-adaptation process (a process that is typically examined using lists with a lower, i.e., 20–33%, proportion of congruent items, see Bugg & Crump, 2012, for a review), this fact should not have prevented a reactive conflict-adaptation process (the main focus of the present experiment) from taking place as well. Finally, the assignment of each set to the MC, MI, or transfer condition was counterbalanced across participants, as was the assignment of the possible color-word combinations in the transfer set.

#### Procedure

Each trial began with a fixation symbol (“+”) displayed for 250 ms in the center of the screen followed by a colored word displayed for 2,000 ms or until the participant’s response, which was recorded with a microphone connected to the testing computer. There was a 750-ms blank screen between trials. Participants were instructed to name the color of the word as quickly and as accurately as possible while ignoring the word itself.

Stimuli were presented in uppercase Courier New font, 14 pt, against a medium-grey background. In line with previous work in our laboratory using the vocal color-word Stroop task (Spinelli & Lupker, 2020, 2021; Spinelli et al., 2020), no feedback was provided. The stimulus presentation involved two equal-sized blocks (192 trials per block) with a self-paced pause in the middle. The order of trials within each block was randomized. Initially, participants performed a practice session involving six trials in which a string of Xs (“XXXX”) was presented in each of the six colors used in the experiment. The experiment was run using DMDX (Forster & Forster, 2003) software.

#### Data treatment and analysis

The waveforms of responses were manually inspected with CheckVocal (Protopapas, 2007) to determine response accuracy and the correct placement of timing marks. Prior to all analyses, invalid trials due to technical failures and responses faster than 300 ms or slower than 2,000 ms (accounting for .9% of the data) were discarded. Prior to the RT analyses, incorrect responses (accounting for 1.9% of the data) were also discarded. Analyses were performed with JASP version 0.14.1 (JASP Team, 2020). For this and the following experiment, the raw data and JASP files used for the analyses are publicly available via the Open Science Framework at https://osf.io/v6h9d/.

Before conducting the main analyses based on the stimuli involving transfer components (i.e., a transfer color or a transfer word, all involving incongruent stimuli), we conducted a classic ISPC analysis based on the stimuli not involving transfer components (stimuli that included both congruent or incongruent conditions) in order to establish the presence of an ISPC effect, a pre-condition for examining the conflict-adaptation processes involved in that effect. This analysis, described in the Online Supplementary Materials (OSM) with its results reported in Table 2, did reveal an ISPC effect in both RTs and error rates, with a larger congruency effect for MC items than for MI items. Therefore, despite the modifications that we applied, our paradigm elicited a regular ISPC effect just as more traditional paradigms do.

**Table 2:** Mean reaction times (RTs) and percentage error rates (and corresponding 95% confidence intervals) for the item-specific proportion-congruent (ISPC) analysis conducted for Experiment 1 (based on stimuli involving non-transfer components)

Congruency	RTs		Error rates	
	MC items	MI items	MC items	MI items
Congruent	681 [659, 704]	702 [674, 729]	.24 [.13, .36]	1.39 [.03, 2.74]
Incongruent	814 [777, 851]	748 [724, 772]	3.82 [1.47, 6.17]	1.69 [1.35, 2.04]
Congruency effect	133	46	3.58	.30

*MC* mostly-congruent, *MI* mostly-incongruent

Following the suggestion of a reviewer of a previous version of this article, the data for the (incongruent) stimuli involving transfer components were analyzed using an ANOVA with Trigger Type (Task-irrelevant vs. Task-relevant) and Congruency Proportion (MC vs. MI) as within-subject factors. (For this and the following experiment, another within-subject factor, Block (First vs. Second), was also included in an additional set of analyses, reported in the OSM, to examine the possibility that conflict-adaptation effects might grow over the course of the experiment (Crump & Milliken, 2009; Jacoby et al., 2003; Spinelli & Lupker, 2020). However, Block did not interact with the other factors in either experiment, all *p*s > .07, and, in general, there was no clear tendency for conflict-adaptation effects to increase later in the experiment).

The purpose of the 2 × 2 ANOVA was to examine, first, whether there was overall evidence for triggering of conflict adaptation by task-irrelevant or task-relevant information (i.e., a main effect of Congruency Proportion). The second purpose was to examine whether the magnitude of this effect was modulated by the nature of the triggering component (i.e., an interaction between Congruency Proportion and Trigger Type). Because, however, our primary interest was in the *existence* of separate triggering processes based on task-irrelevant and task-relevant information rather than their overall impact or relative magnitude, we examined those processes not only in an aggregated fashion (in the ANOVA) but also separately. That is, for the task-irrelevant trigger analysis, we contrasted transfer colors (e.g., yellow) appearing with incongruent MC words (e.g., RED) versus incongruent MI words (e.g., WHITE; see items shaded in light grey in Table 1), whereas for the task-relevant trigger analysis, we contrasted transfer words (e.g., YELLOW) appearing with incongruent MC colors (e.g., red) versus incongruent MI colors (e.g., white; see items shaded in dark grey in Table 1). These contrasts were conducted with one-tailed *t*-tests reflecting the alternative hypothesis that the MC condition would elicit higher RTs and error rates than the MI condition. A Bayesian version of this test was also conducted using JASP’s default settings (van Doorn et al., 2021) to calculate *BF*_+0_, with *BF*_+0_ > 1 suggesting evidence in support of the alternative hypothesis of the presence of the effect *H*_+_ (with the plus in the subscript indicating the directionality of the hypothesis), and *BF*_+0_ < 1 suggesting evidence in support of the null hypothesis of the absence of the effect *H*_0_.

Note that these contrasts aimed to establish the existence of task-irrelevant and task-relevant triggering processes at the group level, not how those processes interact at the individual level. For example, it is possible that, while evidence for task-irrelevant and task-relevant triggering processes would emerge overall, the use of those processes may vary at the individual level, with some individuals, for example, showing a larger effect for the task-irrelevant trigger and a smaller effect for the task-relevant trigger, and vice versa for other individuals, depending on the extent to which each individual relies on one particular stimulus component versus the other as a conflict-adaptation trigger. Although whether task-irrelevant and task-relevant triggering processes would be related at the individual level is an interesting question, it is not one we pursued in the present research.

### Results and discussion

The mean RTs and error rates for the conditions examined (i.e., for stimuli involving transfer components) are presented in Table 3.

**Table 3:** Mean reaction times (RTs) and percentage error rates (and corresponding 95% confidence intervals) in MC and MI conditions for the task-irrelevant and task-relevant trigger types examined in Experiment 1 (based on stimuli involving transfer components)

Trigger Type	RTs (ms)			Error rates (%)		
	MC condition	MI condition	Effect	MC condition	MI condition	Effect
Task-irrelevant (MC words vs. MI words)	792 [764, 820]	790 [763, 818]	2	4.51 [2.85, 6.82]	3.10 [1.69, 4.52]	1.41
Task-relevant (MC colors vs. MI colors)	799 [774, 825]	777 [751, 804]	22	4.20 [3.17, 5.23]	3.23 [2.24, 4.22]	.97

*Note*. The contrast for the task-irrelevant component trigger is based on transfer colors appearing with incongruent MC vs. MI words (see items shaded in light grey in Table 1). The contrast for the task-relevant component trigger is based on transfer words appearing with incongruent MC vs. MI colors (see items shaded in dark grey in Table 1)

*MC* mostly-congruent, *MI* mostly-incongruent

First, as hypothesized, there was a main effect of Congruency Proportion in both RTs and error rates (slower and less accurate responses to MC than MI stimuli overall), *F*(1,71) = 5.94, *MSE* = 1686, *p* = .017, ${\eta}_p^2$ = .077, and *F*(1,71) = 5.45, *MSE* = .002, *p* = .022, ${\eta}_p^2$ = .072, respectively. Second, the triggering component modulated the magnitude of this effect, as in the RTs (but not in the error rates) Congruency Proportion interacted with Trigger Type, *F*(1,71) = 6.91, *MSE* = 1084, *p* = .010, ${\eta}_p^2$ = .089, reflecting a larger Congruency Proportion effect when the triggering component was task-relevant (22 ms) versus task-irrelevant (2 ms; no other effect approached significance in either the RT or error analysis, all *F*s < 1). Third, the Congruency Proportion effect was not ubiquitous, as the one-tailed *t*-tests revealed that the 2-ms effect for the task-irrelevant trigger was non-significant, *t*(71) = .26, *p* = .399, ${\eta}_p^2$ = .001, *BF*_+0_ = .16. In contrast, the 22-ms effect for the task-relevant trigger was significant, *t*(71) = 3.54, *p* < .001, ${\eta}_p^2$ = .150, *BF*_+0_ = 68.04. In contrast, in the error rates, the one-tailed *t*-tests revealed, on one hand, a significant effect for the task-irrelevant trigger, with transfer colors producing more errors when appearing with MC versus MI words (a difference of 1.41%), *t*(71) = 2.15, *p* = .018, ${\eta}_p^2$ = .061, *BF*_+0_ = 2.19,. On the other hand, for the task-relevant trigger, although transfer words did produce numerically more errors when appearing with MC versus MI colors (a difference of .97%), this difference did not reach significance, *t*(71) = 1.38, *p* = .086, ${\eta}_p^2$ = .026, *BF*_+0_ = .59.

Overall, these results suggest that both task-irrelevant and task-relevant components can trigger conflict adaptation. In the RTs, this effect appears stronger when triggered by the task-relevant component; however, the parallel effect failed to emerge in the error rates.

## Experiment 2

Experiment 1 produced evidence that, in the ISPC paradigm, both task-irrelevant and, especially, task-relevant components can trigger conflict adaptation. In Experiment 2, we sought to determine whether these conclusions apply only to the particular stimuli used in the color-word Stroop task (i.e., colors and words) or have wider applicability. To that aim, we created a spatial Stroop task in which the nature of task-relevant information (i.e., one of six directions indicated by an arrow, each requiring a specific response) and task-irrelevant information (i.e., one of six spatial locations where the arrow is displayed) was radically different from Experiment 1.

### Method

#### Participants

Eighty-five students at the University of Western Ontario participated to the experiment, which was conducted remotely, for course credit. After discarding too-fast, too-slow, and incorrect responses (see below), nine participants contributed fewer than 75% of their original observations. These participants were removed from the analyses, leaving 76 participants (43 females and 33 males; seven left-handed, 68 right-handed, and one ambidextrous; age 18–26 years). This sample size exceeds that used in Experiment 1 and, therefore, also has a power of essentially 1 to detect an effect as large as the conflict-adaptation effect reported in Bugg and Hutchison’s (2013) Experiment 3. All participants were native English speakers and had normal or corrected-to-normal vision.

#### Materials

**Fig. 1:**
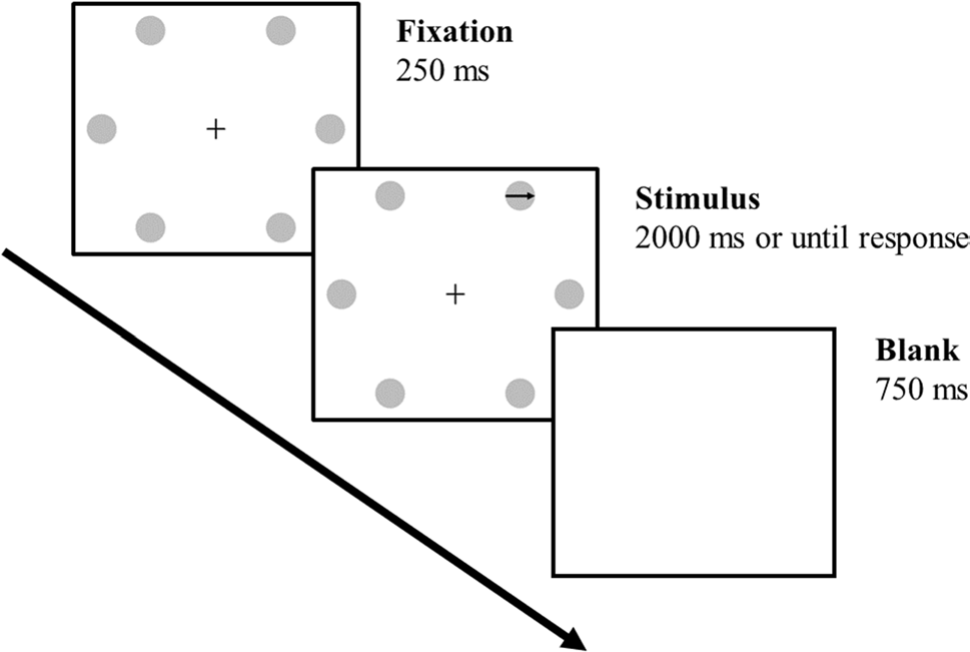
Graphic representation of the materials and procedure in Experiment 2. *Note.* Participants were instructed to respond, as quickly and as accurately as possible, by pressing a button corresponding to the direction the arrow was pointing while ignoring the location in which the arrow was displayed. In this example, an incongruent item is presented in which an east-pointing arrow is displayed in the north-east location. An “east” button response would be required

An illustration of the materials and procedure used in this experiment is presented in Fig. 1 (for a similar task, see Puccioni & Vallesi, 2012). Six medium-grey circles centered on the vertices of an invisible regular hexagon were used to create distractor locations and black arrows pointing in one of six directions (north-east, east, south-east, south-west, west, and north-west, with a 60° angle between each successive direction) were used as targets. The hexagon, which had 222-pixel edges, was arranged so that the bottom and the top edges would be horizontal. As a result, three circles appeared on the right side of the figure and three on the left side. On each trial, an arrow was presented inside one of the circles, with the length of the arrow corresponding to the diameter of the circle (58 pixels). A fixation symbol (“+”) was also displayed in the center of the hexagon. The figures for the stimuli were created with Powerpoint and had a 547-pixel width and a 480-pixel height.

The frequency of arrow-location combinations in one of the two counterbalancings of the experiment is represented in Table 4 (again, in the following, this particular counterbalancing will be used for our examples). Similar to Experiment 1, the stimuli were divided into three sets, with the north-east and south-east locations and the corresponding arrows forming one set, the north-west and south-west locations and the corresponding arrows forming another set, and the east and west locations and the corresponding arrows forming the third set. For each participant, either the north-east/south-east set served as the MC set and the north-west/south-west served as the MI set (the counterbalancing represented in Table 4), or the reverse. In contrast, the east/west set always served as the transfer set. The reason that a simpler counterbalancing scheme was used in this experiment compared to that in Experiment 1 was that, in this experiment, there were two response “modes,” rather than just an oral mode (Lee & Cho, 2013), as responses to north-east-, east-, and south-east-pointing arrows were done with one hand (the right hand), and responses to north-west-, west-, and south-west-pointing arrows were done with the other hand (the left hand; for further details, see below). Because conflict-adaptation processes sometimes do not generalize across response modes (e.g., hands – Kim & Cho, 2014; Lim & Cho, 2018), for each responding hand, in addition to one arrow and one location from the transfer set (e.g., the east arrow/location), only one type of context set (either MC or MI) was used for the other two arrows and two locations (e.g., the north-east and south-east arrows/locations, both MC items). That is, the type of context set used was blocked on the responding hand. Further, only east and west arrows/locations were used for the transfer set because, by doing so, a transfer location was always adjacent to two context locations associated with the same conflict frequency (e.g., the east location was adjacent with the north-east and south-east locations, both MC locations; for evidence that conflict-adaptation processes can transfer from one stimulus location to neighboring locations, see Weidler & Bugg, 2016).

**Table 4:**
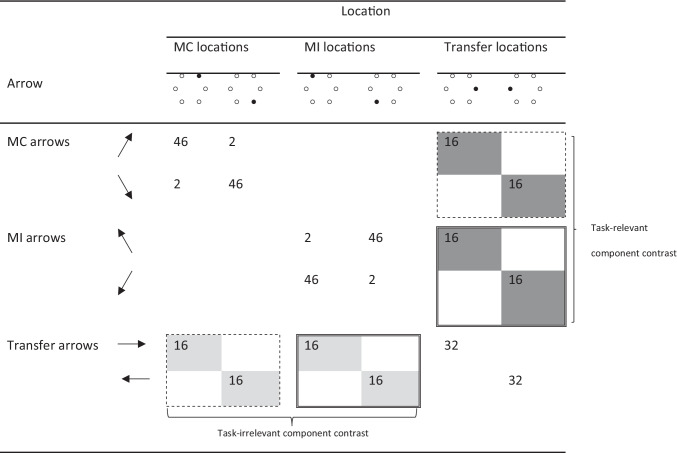
Template for the frequency of arrow-location combinations in Experiment 2

*Note*. The incongruent items shaded in light grey (i.e., the incongruent items appearing with transfer arrows) were used to examine the contrast for the task-irrelevant component triggering of conflict adaptation (i.e., adaptation based on the location). The incongruent items shaded in dark grey (i.e., the incongruent items appearing with transfer locations) were used to examine the contrast for the task-relevant component triggering of conflict adaptation (i.e., adaptation based on the arrow). For each of these contrasts, the items surrounded by a dashed line were used for the MC condition and the items surrounded by a double solid line were used for the MI condition

*MC* mostly-congruent, *MI* mostly-incongruent

The frequency of arrow-location combinations was similar to the frequency of word-color combinations in Experiment 1. Most importantly, the transfer set was arranged so that each arrow in that set (e.g., the east-pointing arrow) appeared 32 times with its congruent location (e.g., the east location), 16 times with one of the (incongruent) MC locations (e.g., the north-east location), and 16 times with one of the (incongruent) MI locations (e.g., the north-west location). These incongruent stimuli, shaded in light grey in Table 4, allowed us to examine task-irrelevant triggering of conflict adaptation because they only differed in task-irrelevant conflict frequency (low for the transfer arrows appearing with MC locations, surrounded by a dashed line in Table 4, vs. high for the transfer arrows appearing with MI locations, surrounded by a double solid line in Table 4).

Note that MC and MI locations were either on the same side as the transfer arrow (thus priming the appropriate response hand) or on the other side (thus priming the inappropriate response hand). However, the side of MC versus MI locations relative to the arrow was counterbalanced across the two transfer arrows. For example, in the version of the experiment presented in Table 4, the east-pointing arrow appeared with a same-side MC location (i.e., the north-east location) and an other-side MI location (i.e., the north-west location), whereas the west-pointing arrow appeared with an other-side MC location (i.e., the south-east location) and a same-side MI location (i.e., the south-west location).

The transfer set was also arranged so that each location in that set (e.g., the east location) appeared 32 times with its congruent arrow (e.g., the east-pointing arrow), 16 times with one of the (incongruent) MC arrows (e.g., the north-east-pointing arrow), and 16 times with one of the (incongruent) MI arrows (e.g., the north-west-pointing arrow). These incongruent stimuli, shaded in dark grey in Table 4, served to examine task-relevant triggering of conflict adaptation because they only differed in task-relevant conflict frequency (low for the transfer locations appearing with MC arrows, surrounded by a dashed line in Table 4, vs. high for the transfer locations appearing with MI arrows, surrounded by a double solid line in Table 4).

Again, MC and MI arrows were either on the same side as the transfer location (thus requiring a response from the same hand primed by the location) or on the other side (thus requiring a response from the other hand than that primed by the location). In this case as well, however, the side of MC versus MI arrows relative to the location was counterbalanced across the two transfer locations. For example, in the version of the experiment presented in Table 4, the east location appeared with a same-side MC arrow (i.e., the north-east-pointing arrow) and an other-side MI arrow (i.e., the north-west-pointing arrow), whereas the west location appeared with an other-side MC arrow (i.e., the south-east-pointing arrow) and a same-side MI arrow (i.e., the south-west-pointing arrow). Overall, as in Experiment 1, there were 384 items (160 congruent and 224 incongruent).

#### Procedure

An illustration of the materials and procedure is presented, as noted, in Fig. 1. Each trial began with a fixation figure in which the six circles, all empty, were displayed for 250 ms. Subsequently, an arrow was displayed in one of the circles for 2,000 ms or until the participant’s response. In both displays, a fixation symbol (“+”) was displayed in the center of the invisible hexagon. The hexagon itself was centered on the screen. Finally, there was a 750-ms blank screen between trials. Participants were instructed to respond as quickly and as accurately as possible by pressing the button corresponding to the direction of the arrow while ignoring the location at which the arrow was being displayed. Specifically, they were instructed to press the U-key with the right middle finger for “north-east” responses, the J-key with the right index finger for “east” responses, the N-key with the right thumb for “south-east” responses, the V-key with the left thumb for “south-west” responses, the F-key with the left index finger for “west” responses, and the T-key with the left middle finger for “north-west” responses. Note that in common keyboard layouts, these key positions are spatially compatible with the arrows and locations used. This compatibility is an important determinant of the size of congruency effects in spatial Stroop tasks and parallels the compatibility existing between words and vocal responses in the color-word Stroop task (Lu & Proctor, 2001). The stimuli were presented against a white background in a full-screen browser window. The stimuli were presented in two equal-sized blocks (192 trials per block) with a self-paced pause in the middle. The order of trials within each block was randomized.

Initially, participants performed a practice session including two blocks. The first block consisted of 30 trials in which a single circle was presented in the center of the screen. The circle was empty for 250 ms and then an arrow appeared inside it for 2,000 ms or until the participant’s response. The second block consisted of 72 trials, with the same materials and procedure as in the experimental session. The frequency of arrow/location combinations also mirrored that of the following experimental session. A longer practice session was included in this experiment to allow participants to familiarize themselves with the stimulus-response mappings.

As in Experiment 1, no feedback was provided in the experimental session. However, feedback was provided in the practice session in order to further facilitate learning of the stimulus-response mappings. In this session, after the stimulus display and before the blank screen, the feedback message “Correct” was displayed in green if the response made was correct, “Wrong” in red if the response was incorrect, and “No response,” also in red, if no response was made. All feedback messages were displayed in 36 pt Courier New font for 500 ms. The experiment was run using the jsPsych (de Leeuw, 2015) JavaScript library.

#### Data treatment and analysis

Prior to all analyses, invalid trials due to technical failures and responses faster than 300 ms or slower than 2,000 ms (accounting for 1% of the data) were discarded. Prior to the RT analyses, incorrect responses (accounting for 4.6% of the data) were also discarded. The analyses were conducted in a similar fashion to those in Experiment 1. For this experiment as well, a classic ISPC analysis (reported in the OSM) based on the stimuli not involving transfer components was conducted before the main analyses to establish the presence of an ISPC effect. As illustrated in Table 5, that effect did emerge clearly in both RTs and error rates.

**Table 5:** Mean reaction times (RTs) and percentage error rates (and corresponding 95% confidence intervals) for the item-specific proportion-congruent (ISPC) analysis conducted for Experiment 2 (based on stimuli involving non-transfer components)

Congruency	RTs		Error rates	
	MC items	MI items	MC items	MI items
Congruent	594 [570, 617]	627 [600, 655]	1.28 [.93, 1.62]	1.32 [.03, 2.60]
Incongruent	731 [698, 764]	697 [671, 722]	9.54 [5.27, 13.81]	3.80 [3.16, 4.44]
Congruency effect	137	70	8.26	2.48

*MC* mostly-congruent, *MI* mostly-incongruent

### Results and discussion

The mean RTs and error rates for the conditions examined (i.e., for stimuli involving transfer components) are presented in Table 6.

**Table 6:** Mean reaction times (RTs) and percentage error rates (and corresponding 95% confidence intervals) in the MC and MI conditions for the task-irrelevant and task-relevant trigger types examined in Experiment 2 (based on stimuli involving transfer components)

Trigger type	RTs (ms)			Error rates (%)		
	MC condition	MI condition	Effect	MC condition	MI condition	Effect
Task-irrelevant (MC locations vs. MI locations)	696 [671, 721]	696 [671, 720]	0	11.45 [9.39, 13.51]	7.68 [6.20, 9.15]	3.77
Task-relevant (MC arrows vs. MI arrows)	734 [706, 763]	717 [691, 743]	17	10.48 [8.36, 12.60]	7.94 [6.34, 9.55]	2.54

*Note*. The contrast for the task-irrelevant trigger is based on transfer arrows appearing with incongruent MC vs. MI locations (see items shaded in light grey in Table 4). The contrast for the task-relevant trigger is based on transfer locations appearing with incongruent MC vs. MI arrows (see items shaded in dark grey in Table 4)

*MC* mostly-congruent, *MI* mostly-incongruent

In the RTs (but not in the error rates), there was a main effect of Trigger Type, *F*(1,75) = 16.44, *MSE* = 4120, *p* < .001, ${\eta}_p^2$ = .180, reflecting overall slower responses to the stimuli involved in the task-relevant versus task-irrelevant contrast, presumably because responding to the arrows used in the former contrast (i.e., north-east, south-east, south-west, and north-west responses) was harder than responding to the arrows used in the latter contrast (i.e., east and west responses; see Table 4). More relevant to our hypotheses, first, there was a main effect of Congruency Proportion in the error rates (less accurate responses to MC than MI stimuli overall), *F*(1,75) = 19.28, *MSE* = .004, *p* < .001, ${\eta}_p^2$ = .204, but not in the RTs, *F*(1,75) = 2.74, *MSE* = 2154, *p* = .102, ${\eta}_p^2$ = .035. Second, although in the RTs there was a numerical tendency for a larger Congruency Proportion effect when the triggering component was task-relevant, the interaction did not reach significance in either the RTs, *F*(1,75) = 3.47, *MSE* = 2154, *p* = .066, ${\eta}_p^2$ = .044, or the error rates, *F* < 1.

Third, separate examinations of the task-irrelevant and task-relevant contrasts revealed that, in the former, RTs were essentially equivalent for transfer arrows (e.g., the east-pointing arrow) appearing in incongruent MC locations (e.g., the north-east location) versus MI locations (e.g., the north-west location; see items shaded in light grey in Table 4), *t*(75) = .06, *p* = .477, ${\eta}_p^2$ < .001, *BF*_+0_ = .13. In contrast, in the task-relevant contrast, transfer locations (e.g., the east location) were slower when appearing with incongruent MC arrows (e.g., the north-east-pointing arrow) versus MI arrows (e.g., the north-west-pointing arrow; see items shaded in dark grey in Table 4), a significant 17-ms difference, *t*(75) = 2.59, *p* = .006, ${\eta}_p^2$ = .082, *BF*_+0_ = 5.54.

In the error rates, on the other hand, MC stimuli produced significantly more errors than MI stimuli in both the task-irrelevant contrast (a difference of 3.77%), *t*(75) = 3.83, *p* < .001, ${\eta}_p^2$ = .163, *BF*_+0_ = 164.05, and the task-relevant contrast (a difference of 2.54%), *t*(75) = 2.37, *p* = .010, ${\eta}_p^2$ = .070, *BF*_+0_ = 3.43.

Overall, similar to Experiment 1, these results suggest that conflict adaptation can be triggered by both task-irrelevant and task-relevant components, even though there was no evidence that the task-irrelevant component had any impact in the RT analysis.

## General discussion

Although the ISPC paradigm (Jacoby et al., 2003) has attracted considerable research interest for examining the process of adjusting control in a reactive, item-specific fashion, recent findings suggest that this process may be engaged only in situations that prevent contingency learning from concurrently being engaged, situations in which the task-relevant stimulus component is typically the only viable conflict-adaptation trigger (e.g., Chiu et al., 2017). However, conflict-adaptation processes may also be (1) engaged when contingency learning is also a viable option (Spinelli & Lupker, 2020) and (2) triggered by the task-irrelevant component (Bugg & Hutchison, 2013), as is possible in the original ISPC paradigm in which both components are equally strong cues to conflict frequency.

In order to examine the question of which component triggers conflict adaptation without imposing limitations on the processes potentially contributing to the ISPC effect, we modified the original ISPC paradigm to allow a dissociation of task-relevant versus task-irrelevant triggering processes and to eliminate a contingency-learning explanation of those effects. In both a color-word Stroop task and a spatial Stroop task, we obtained evidence supporting the presence of both processes, as performance was overall worse for incongruent MC stimuli compared with incongruent MI stimuli matched on all variables of interest. More specifically, in the key contrasts, incongruent MC task-relevant stimuli elicited slower RTs (in Experiments 1 and 2) and more errors (in Experiment 2) than did incongruent MI task-relevant stimuli. Similarly, in both experiments, incongruent MC task-irrelevant stimuli produced more errors than did incongruent MI task-irrelevant stimuli, although the RTs for the two types of stimuli were comparable in both cases. These results suggest triggering processes whereby, on one hand, recognition of MC task-relevant or task-irrelevant information would induce a state of lower selectivity (i.e., less adaptation to potential conflict) in which task-irrelevant information would have more influence in the selection process, even if the stimulus is actually incongruent. On the other hand, recognition of MI task-relevant or task-irrelevant information would induce a state of higher selectivity facilitating selection of task-relevant information in that situation.

Although the present results show that conflict adaptation may be triggered by both task-irrelevant and task-relevant information, they reveal little about the specific processes that allow individuals to avoid errors and resolve interference in the tasks. One potential clue to the nature of those processes is that, in the RTs, the Congruency Proportion effect for the task-relevant trigger tended to be larger than the (null) Congruency Proportion effect for the task-irrelevant trigger. Because RT differences for incongruent stimuli are typically interpreted as reflecting differences in interference-resolution processes (Kane & Engle, 2003), the RT pattern we obtained may suggest that interference from task-irrelevant information is resolved more efficiently for MI than MC stimuli only when conflict adaptation is triggered by task-relevant information. For example, recognizing an MI color, compared to an MC color, would not only prevent, in most cases, the erroneous selection of the incongruent word (resulting in fewer errors), but it would also aid in resolving the interference created by that word (resulting in faster RTs). In contrast, recognizing an MI word, compared to an MC word, would only prevent, in most cases, the erroneous selection of the incongruent word.

A potential problem with these ideas is that the error-rate pattern went in the opposite direction, with the Congruency Proportion effect being numerically *smaller* for the task-relevant versus task-irrelevant trigger, suggesting a potential speed-accuracy trade-off. Although Spearman correlations between overall RTs and error rates for each participant revealed no evidence of a speed-accuracy trade-off in either Experiment 1, *r*(72) = .04, *p* = .730, or Experiment 2, *r*(72) = -.10, *p* = .380, a common way to address ambiguities between RTs and error rates is to combine the two variables. If conflict adaptation triggered by task-relevant information affects two processes (i.e., interference resolution, typically reflected in RTs, and error avoidance, typically reflected in error rates), it would seem to follow that the associated effect should be larger in a combined measure of RTs and error rates compared with the effect associated with the task-irrelevant trigger, which may only affect one process (i.e., error avoidance).

A traditional way to combine speed and accuracy is to use Inverse-Efficiency Scores (IES) obtained by dividing, for each participant, the mean RT within each condition by its respective accuracy (i.e., the proportion of correct responses; Townsend & Ashby, 1983). A more recent, and potentially more efficient, combination of the two measures is the Balanced Integration Score (BIS) obtained by standardizing the mean RT and accuracy of each participant and subtracting one standardized measure from the other (Liesefeld & Janczyk, 2019). Re-analyses using IES and BIS, reported in the OSM, showed a Congruency Proportion effect but little or no evidence in either experiment for an interaction between that factor and Trigger Type. Further, as illustrated in Table 7, when all analyses (with RTs, error rates, IES, and BIS) were repeated using Bayesian ANOVAs, the Bayes factor representing the evidence in favor of the presence (*H*_1_) versus the absence (*H*_0_) of the interaction, *BF*_10_, supported the absence in all cases except for RTs in Experiment 1 (and even in that case, the evidence in favor of the presence was weak). Thus, overall, there is little evidence in the present data that conflict-adaptation processes triggered by task-irrelevant versus task-relevant information differ in any substantial way.

**Table 7:** Bayes factor (BF_10_) values in favor of the presence (alternative hypothesis H_1_) versus the absence (null hypothesis H_0_) of the interaction between congruency proportion and trigger type for stimuli involving transfer components in Experiments 1 and 2 for all dependent variables examined

Experiment	Dependent variable			
	RT	ER	IES	BIS
Experiment 1	1.77	.18	.25	.18
Experiment 2	.42	.24	.28	.15

*Note*. For each experiment and dependent variable, *BF*_10_ was obtained by comparing the model including the interaction between Congruency Proportion and Trigger Type and the model that only included the main effects of Congruency Proportion and Trigger Type using JASP’s default settings

*RT* response time, *ER* error rate, *IES* inverse-efficiency score, *BIS* balanced integration score

The present experiments, in any case, were not set up to examine the impact of conflict adaptation on error avoidance, interference resolution, or the exact mechanism (inhibition of task-irrelevant information vs. enhancement of task-relevant information) whereby conflict is eventually resolved. On this note, imposing a response deadline and analyzing the data with a procedure capable of dissociating the independent contributions of word-reading and color-naming processing to color-word Stroop performance, Jacoby et al. (2003) concluded that ISPC manipulations mainly modulate task-irrelevant processing (e.g., inhibiting word reading when an MI stimulus is recognized; but see Egner & Hirsch (2005) for evidence from another paradigm that conflict-adaptation processes may actually modulate task-*relevant* processing). A manipulation similar to that in the present ISPC paradigm would permit an examination of whether this conclusion would apply to conflict-adaptation processes triggered by both task-irrelevant versus task-relevant information, a distinction that was not possible in Jacoby et al.’s (2003) original paradigm.

In summary, the present data indicate that it is unlikely that conflict adaptation contributes to the ISPC effect only under select circumstances. Because in the present experiments all types of processes (i.e., task-irrelevant and task-relevant triggering of conflict adaptation, as well as contingency learning) could be used and yet evidence for both task-irrelevant and task-relevant triggering emerged, it seems likely that all those processes are often engaged simultaneously in any ISPC paradigms that allow their use. This idea does not imply, of course, that manipulations designed to bias use of one of those processes would be ineffective at doing so or that all possible processes potentially contributing to the ISPC effect must be engaged in those ISPC paradigms as well. Moving forward, however, dissociation procedures such as those used in the present experiments will be needed if the specific processes involved in more neutral ISPC paradigms are to be successfully understood.

## Supplementary Information

ESM 1 (DOCX 43.7 kb)
