# Supplementary material for: Both task-irrelevant and task-relevant information trigger reactive conflict adaptation in the item-specific proportion-congruent paradigm
Source: Psychon Bull Rev. 2022 Jun 29;29(6):2133–45. doi: 10.3758/s13423-022-02138-5 (PMC9722811; doi:10.3758/s13423-022-02138-5)
Supplement: Supplementary file 1 — (DOCX 43.7 kb) [file 13423_2022_2138_MOESM1_ESM.docx]

**Supplementary Materials**

Classic ISPC Analysis

In the original ISPC paradigm (Jacoby et al., 2003), congruency effects are larger for MC items than for MI items. In that paradigm, the MC items typically combine an MC task-irrelevant stimulus component (e.g., the word RED presented most often with the congruent color red) and an MC task-relevant component (e.g., the color red presented most often with the congruent word RED); similarly, the MI items typically combine an MI task-irrelevant component (e.g., the word WHITE presented most often with the incongruent color green) and an MI task-relevant component (e.g., the color white presented most often with the incongruent word GREEN).

To determine if our modified ISPC paradigm would also elicit an ISPC effect, for RTs and error rates in both Experiments 1 and 2, we ran an analysis on the items in our paradigm that were most similar to those used in the standard paradigm. Specifically, we contrasted congruency effects for stimuli that combined non-transfer components, i.e., for stimuli that combined an MC task-irrelevant component and an MC task-relevant component (the MC items, e.g., RED, BLUE, and the corresponding colors in Experiment 1; see Table 1) and for stimuli that combined an MI task-irrelevant component and an MI task-relevant component (the MI items, e.g., WHITE, GREEN, and the corresponding colors in Experiment 1). That is, for those items, an ANOVA was run with Congruency (Congruent vs. Incongruent) and Congruency Proportion (MC vs. MI) as within-subject factors. The results for Experiments 1 and 2 are reported in separate sections below.

*Experiment 1*

The mean RTs and error rates for this analysis are presented in Table 2 in the main text and reproduced here in Table S1. In the RTs, this analysis revealed not only main effects of Congruency (faster responses to congruent than incongruent items), *F*(1,71) = 101.95, *MSE* = 5656, *p* < .001, $\eta_{p}^{2}$ = .589, and Congruency Proportion (slower responses to MC than MI items overall), *F*(1,71) = 7.00, *MSE* = 5171, *p* = .010, $\eta_{p}^{2}$ = .090, but also an interaction between the two, *F*(1,71) = 33.67, *MSE* = 3924, *p* < .001, $\eta_{p}^{2}$ = .322. This interaction reflected the typical ISPC effect, with a larger congruency effect for MC items (133 ms) than for MI items (46 ms). The Bayes Factor for the comparison between the model with the interaction (interpreted as the alternative hypothesis *H_1_*) and the model without it (interpreted as the null hypothesis *H_0_*) was *BF*_10_ = 25346.64 ± 3.69%. In Jeffreys’s (1961) classification scheme (as reported in adjusted form by Lee and Wagenmakers, 2013), this value would suggest “extreme” evidence for the presence of the interaction.

Similarly, for the error rates, the ISPC analysis revealed a main effect of Congruency (more accurate responses to congruent than incongruent items), *F*(1,71) = 7.54, *MSE* = .004, *p* = .008, $\eta_{p}^{2}$ = .096, albeit not Congruency Proportion, *F*(1,71) = .47, *MSE* = .004, *p* = .497, $\eta_{p}^{2}$ = .007. The Congruency by Congruency Proportion interaction was significant, *F*(1,71) = 6.02, *MSE* = .003, *p* = .017, $\eta_{p}^{2}$ = .078, indicating a larger congruency effect for MC items (3.58%) than for MI items (.30%), the typical pattern of the ISPC effect. On the other hand, the Bayes Factor, *BF*_10_ = 2.77 ± 3.91%, suggested only “anecdotal” evidence for the presence of the interaction. In any case, both RTs and error rates produced an ISPC effect, suggesting that our ISPC paradigm remained valid despite the modifications that we applied.

Table S1

*Mean RTs and Percentage Error Rates (and Corresponding 95% Confidence Intervals) for the ISPC Analysis Conducted for Experiment 1 (Based on Stimuli Involving Non-Transfer Components)*

|  | RTs | | Error rates | |
| --- | --- | --- | --- | --- |
| Congruency | MC items | MI items | MC items | MI items |
| Congruent | 681 [659, 704] | 702 [674, 729] | .24 [.13, .36] | 1.39 [.03, 2.74] |
| Incongruent | 814 [777, 851] | 748 [724, 772] | 3.82 [1.47, 6.17] | 1.69 [1.35, 2.04] |
| Congruency Effect | 133 | 46 | 3.58 | .30 |

*Experiment 2*

The mean RTs and error rates for this analysis are presented in Table 5 in the main text and reproduced here in Table S2. In the RTs, there was a main effect of Congruency (faster responses to congruent than incongruent items), *F*(1,75) = 199.50, *MSE* = 4065, *p* < .001, $\eta_{p}^{2}$ = .727, but no main effect of Congruency Proportion, *F*(1,75) < .01, *MSE* = 3364, *p* = .961, $\eta_{p}^{2}$ < .001. The two factors interacted, however, *F*(1,75) = 26.40, *MSE* = 3380, *p* < .001, $\eta_{p}^{2}$ = .260. This interaction reflected the typical ISPC effect, with a larger congruency effect for MC items (137 ms) than for MI items (70 ms). The Bayes Factor for the comparison between the model with the interaction and the model without it was *BF*_10_ = 10057.40 ± 3.11%, indicating “extreme” evidence for the presence of the interaction.

In the error rates, there were main effects of Congruency (more accurate responses to congruent than incongruent items), *F*(1,75) = 20.93, *MSE* = .010, *p* < .001, $\eta_{p}^{2}$ = .218, as well as Congruency Proportion (less accurate responses to MC than MI items overall), *F*(1,75) = 6.02, *MSE* = .010, *p* = .016, $\eta_{p}^{2}$ = .074. The Congruency by Congruency Proportion interaction was also significant, *F*(1,75) = 7.86, *MSE* = .008, *p* = .006, $\eta_{p}^{2}$ = .095, indicating a larger congruency effect for MC items (8.26%) than for MI items (2.48%), the typical pattern of the ISPC effect. The Bayes Factor, *BF*_10_ = 4.29 ± 5.16%, indicated “moderate” evidence for the presence of the interaction. In sum, in Experiment 2 as well as in Experiment 1, both RTs and error rates produced an ISPC effect, suggesting that the validity of our modified ISPC paradigm is not restricted to the color-word Stroop task but extends to the spatial Stroop task as well.

Table S2

*Mean RTs and Percentage Error Rates (and Corresponding 95% Confidence Intervals) for the ISPC Analysis Conducted for Experiment 2 (Based on Stimuli Involving Non-Transfer Components)*

|  | RTs | | Error rates | |
| --- | --- | --- | --- | --- |
| Congruency | MC items | MI items | MC items | MI items |
| Congruent | 594 [570, 617] | 627 [600, 655] | 1.28 [.93, 1.62] | 1.32 [.03, 2.60] |
| Incongruent | 731 [698, 764] | 697 [671, 722] | 9.54 [5.27, 13.81] | 3.80 [3.16, 4.44] |
| Congruency Effect | 137 | 70 | 8.26 | 2.48 |

Block Analysis

In this analysis, the RTs and error rates for the (incongruent) stimuli involving transfer components in Experiments 1 and 2 were analyzed using an ANOVA with Block (First vs. Second) as a within-subject factor as well as Trigger Type (Task-irrelevant vs. Task-relevant) and Congruency Proportion (MC vs. MI), the (within-subject) factors included in the analyses reported in the main text. As explained in the main text, the inclusion of the Block factor was meant to account for the possibility that conflict-adaptation effects might grow over the course of the experiment (Crump & Milliken, 2009; Spinelli & Lupker, 2020).

*Experiment 1*

The mean RTs and error rates for this analysis are presented in Table S3. In the RTs, there was a main effect of Congruency Proportion (slower responses to MC than MI stimuli overall), *F*(1,71) = 6.03, *MSE* = 3406, *p* = .017, $\eta_{p}^{2}$ = .078, and an interaction between Congruency Proportion and Trigger Type, *F*(1,71) = 7.34, *MSE* = 2148, *p* = .008, $\eta_{p}^{2}$ = .094, reflecting a larger effect of Congruency Proportion when the triggering component was task-relevant vs. task-irrelevant. There was also a marginal interaction between Congruency Proportion, Trigger Type, and Block, *F*(1,71) = 3.26, *MSE* = 1494, *p* = .075, $\eta_{p}^{2}$ = .044, reflecting the fact that while the effect of Congruency Proportion for the task-irrelevant trigger tended to increase from the first block, in which it was negative (-5 ms), to the second block (9 ms), the effect of Congruency Proportion for the task-relevant trigger showed the opposite tendency, decreasing from 27 ms in the first block to 18 ms in the second block. No other effect approached significance, all *F*s < 1.

In the error rates, the only significant effect was the main effect of Congruency Proportion (less accurate responses to MC than MI stimuli overall), *F*(1,71) = 5.42, *MSE* = .004, *p* = .023, $\eta_{p}^{2}$ = .071, all other *p*s > .2.

Table S3

*Mean RTs and Percentage Error Rates (and Corresponding 95% Confidence Intervals) in MC and MI Conditions for the Task-Irrelevant and Task-Relevant Trigger Types Examined in the First and Second Blocks in Experiment 1 (Based on Stimuli Involving Transfer Components)*

|  | RTs (ms) | |  | Error rates (%) | |  |
| --- | --- | --- | --- | --- | --- | --- |
| Trigger Type | MC condition | MI condition | Effect | MC condition | MI condition | Effect |
| *Block 1* |  |  |  |  |  |  |
| Task-irrelevant  (MC words vs. MI words) | 792 [763, 820] | 797 [768, 826] | -5 | 4.81 [2.99, 6.64] | 2.75 [1.18, 4.31] | 2.06 |
| Task-relevant (MC colors vs. MI colors) | 804 [777, 830] | 777 [750, 803] | 27 | 3.87 [2.63, 5.12] | 3.10 [2.03, 4.16] | .77 |
| *Block 2* |  |  |  |  |  |  |
| Task-irrelevant  (MC words vs. MI words) | 793 [762, 823] | 784 [754, 814] | 9 | 4.18 [2.32, 6.05] | 3.48 [1.97, 4.99] | .70 |
| Task-relevant (MC colors vs. MI colors) | 796 [768, 823] | 778 [749, 807] | 18 | 4.54 [3.18, 5.91] | 3.36 [1.88, 4.84] | 1.18 |

*Note*. The contrast for the task-irrelevant trigger is based on transfer colors appearing with incongruent MC vs. MI words (see items shaded in light grey in Table 1 in the main text). The contrast for the task-relevant trigger is based on transfer words appearing with incongruent MC vs. MI colors (see items shaded in dark grey in Table 1 in the main text).

*Experiment 2*

The mean RTs and error rates for this analysis are presented in Table S4. In the RTs, there was a main effect of Trigger Type, *F*(1,75) = 16.58, *MSE* = 8246, *p* < .001, $\eta_{p}^{2}$ = .181, reflecting overall slower responses to the stimuli involved in the task-relevant vs. task-irrelevant contrast. As noted in the main text, it can be presumed that producing the responses required by the arrows used in the former contrast (i.e., north-east, south-east, south-west, and north-west responses) was harder than producing the responses required by the arrows used in the latter contrast (i.e., east and west responses; see Table 4). There was also a main effect of Block (slower responses in the first vs. second block, a practice effect), *F*(1,75) = 58.69, *MSE* = 6219, *p* < .001, $\eta_{p}^{2}$ = .435. MC stimuli were also numerically slower than MI stimuli overall, but the main effect of Congruency Proportion did not reach significance, *F*(1,75) = 2.49, *MSE* = 4211, *p* = .119, $\eta_{p}^{2}$ = .032. In fact, no other effect reached significance, all *p*s > .10.

In the error rates, there was no main effect of Trigger Type, *F*(1,75) = 2.49, *MSE* = 4211, *p* = .119, $\eta_{p}^{2}$ = .032, but there were main effects of Block (less accurate responses in the first vs. second block, a practice effect), *F*(1,75) = 6.80, *MSE* = .006, *p* = .011, $\eta_{p}^{2}$ = .083, and Congruency Proportion (less accurate responses to MC than MI stimuli), *F*(1,75) = 19.32, *MSE* = .008, *p* < .001, $\eta_{p}^{2}$ = .205. No other effect reached significance, all *F*s < 1.

Table S4

*Mean RTs and Percentage Error Rates (and Corresponding 95% Confidence Intervals) in MC and MI Conditions for the Task-Irrelevant and Task-Relevant Trigger Types Examined in the First and Second Blocks in Experiment 2 (Based on Stimuli Involving Transfer Components)*

|  | RTs (ms) | |  | Error rates (%) | |  |
| --- | --- | --- | --- | --- | --- | --- |
| Trigger Type | MC condition | MI condition | Effect | MC condition | MI condition | Effect |
| *Block 1* |  |  |  |  |  |  |
| Task-irrelevant  (MC locations vs. MI locations) | 720 [694, 747] | 723 [696, 750] | -3 | 11.70 [9.08, 14.31] | 8.91 [6.78, 11.04] | 2.79 |
| Task-relevant (MC arrows vs. MI arrows) | 758 [727, 788] | 740 [708, 771] | 18 | 11.42 [8.78, 14.07] | 8.66 [6.63, 10.70] | 2.76 |
| *Block 2* |  |  |  |  |  |  |
| Task-irrelevant  (MC locations vs. MI locations) | 672 [646, 698] | 668 [643, 693] | 4 | 11.16 [8.80, 13.53] | 6.35 [4.74, 7.96] | 4.81 |
| Task-relevant (MC arrows vs. MI arrows) | 707 [679, 740] | 697 [671, 722] | 10 | 9.54 [7.52, 11.57] | 7.32 [5.47, 9.27] | 2.22 |

*Note*. The contrast for the task-irrelevant trigger is based on transfer arrows appearing with incongruent MC vs. MI locations (see items shaded in light grey in Table 4 in the main text). The contrast for the task-relevant trigger is based on transfer locations appearing with incongruent MC vs. MI arrows (see items shaded in dark grey in Table 4 in the main text).

Analyses Using Combined Latency and Accuracy Measures

In these analyses, the RTs and error rates for the (incongruent) stimuli involving transfer components in Experiments 1 and 2 were combined to produce Inverse-Efficiency Score (IES; Townsend & Ashby, 1983) and Balanced Integration Score (BIS; Liesefeld & Janczyk, 2019) values. IES values were obtained by dividing, for each participant, the mean RT within each condition by its respective accuracy (i.e., the proportion of correct responses). BIS values were obtained by standardizing, for each participant, the mean RT and the mean accuracy in each condition across all RTs and accuracy values, respectively, used in the analysis, and subtracting the standardized accuracy from the standardized RT (i.e., the same calculation used by Liesefeld & Janczyk, 2019, with the exception that they subtracted the standardized RT from the standardized accuracy; we did the reverse to maintain the directionality of the effects of the other measures, so that that lower values correspond to better performance). Both IES and BIS were used because, whereas the former is the procedure traditionally used to combine latency and accuracy measures, its effectiveness at summarizing findings across those measures is not without issues (Bruyer & Brysbaert, 2011), issues that do not appear to affect the more recently introduced BIS to the same degree (Liesefeld & Janczyk, 2019).

IES and BIS values were analyzed in the same way as in the main text, i.e., using an ANOVA with Trigger Type (Task-irrelevant vs. Task-relevant) and Congruency Proportion (MC vs. MI) as within-subject factors. As noted in the main text, these analyses helped address the potential ambiguity between RTs and error rates concerning which component plays the stronger role in triggering conflict adaptation. The mean IES values for Experiments 1 and 2 are presented in Table S5. The mean BIS values for Experiments 1 and 2 are presented in Table S6.

Table S5

*Mean IES Values (and Corresponding 95% Confidence Intervals) in the MC and MI Conditions for the Task-Irrelevant and Task-Relevant Trigger Types Examined in Experiments 1 and 2 (Based on Stimuli Involving Transfer Components)*

|  | Experiment 1 | |  | Experiment 2 | |  |
| --- | --- | --- | --- | --- | --- | --- |
| Trigger Type | MC condition | MI condition | Effect | MC condition | MI condition | Effect |
| Task-irrelevant  (MC locations vs. MI locations) | 836 [800, 873] | 820 [786, 855] | 16 | 796 [759, 833] | 757 [728, 785] | 39 |
| Task-relevant (MC arrows vs. MI arrows) | 836 [807, 866] | 805 [776, 834] | 31 | 836 [801, 872] | 780 [750, 809] | 56 |

*Note*. For Experiment 1, the contrast for the task-irrelevant trigger is based on transfer colors appearing with incongruent MC vs. MI words (see items shaded in light grey in Table 1 in the main text), whereas the contrast for the task-relevant trigger is based on transfer words appearing with incongruent MC vs. MI colors (see items shaded in dark grey in Table 1 in the main text). For Experiment 2, the contrast for the task-irrelevant trigger is based on transfer arrows appearing with incongruent MC vs. MI locations (see items shaded in light grey in Table 4 in the main text), whereas the contrast for the task-relevant trigger is based on transfer locations appearing with incongruent MC vs. MI arrows (see items shaded in dark grey in Table 4 in the main text).

Table S6

*Mean BIS Values (and Corresponding 95% Confidence Intervals) in the MC and MI Conditions for the Task-Irrelevant and Task-Relevant Trigger Types Examined in Experiments 1 and 2 (Based on Stimuli Involving Transfer Components)*

|  | Experiment 1 | |  | Experiment 2 | |  |
| --- | --- | --- | --- | --- | --- | --- |
| Trigger Type | MC condition | MI condition | Effect | MC condition | MI condition | Effect |
| Task-irrelevant  (MC locations vs. MI locations) | .154 [-.234, .542] | -.113 [-.465, .238] | .267 | .124 [-.218, .466] | -.343 [-.613, -.074] | .467 |
| Task-relevant (MC arrows vs. MI arrows) | .163 [-.131, .458] | -.204 [-.506, .098] | .367 | .342 [-.015, .698] | -.122 [-.432, .188] | .464 |

*Note*. For Experiment 1, the contrast for the task-irrelevant trigger is based on transfer colors appearing with incongruent MC vs. MI words (see items shaded in light grey in Table 1 in the main text), whereas the contrast for the task-relevant trigger is based on transfer words appearing with incongruent MC vs. MI colors (see items shaded in dark grey in Table 1 in the main text). For Experiment 2, the contrast for the task-irrelevant trigger is based on transfer arrows appearing with incongruent MC vs. MI locations (see items shaded in light grey in Table 4 in the main text), whereas the contrast for the task-relevant trigger is based on transfer locations appearing with incongruent MC vs. MI arrows (see items shaded in dark grey in Table 4 in the main text).

*Experiment 1*

Using IES, the only significant effect was that of Congruency Proportion (higher scores for MC than MI stimuli overall), *F*(1,71) = 8.65, *MSE* = 4551, *p* = .004, $\eta_{p}^{2}$ = .109. Although the effect of Congruency Proportion for the task-irrelevant trigger (16) did not reach significance in a one-tailed *t*-test, *t*(71) = 1.57, *p* = .060, $\eta_{p}^{2}$ = .034, *BF*_+0_ = .78, and was smaller than for the task-relevant trigger (31, which was significant in a one-tailed *t*-test, *t*(71) = 2.99, *p* = .002, $\eta_{p}^{2}$ = .112, *BF*_+0_ = 14.90), Congruency Proportion and Trigger Type did not interact, *F*(1,71) = 1.48, *MSE* = 2932, *p* = .229, $\eta_{p}^{2}$ = .020. There was no main effect of Trigger Type either, *F* < 1.

Similarly, using BIS, the only significant effect was that of Congruency Proportion (higher scores for MC than MI stimuli overall), *F*(1,71) = 8.25, *MSE* = .881, *p* = .005, $\eta_{p}^{2}$ = .105. There was neither a main effect of Trigger Type nor an interaction between Congruency Proportion and Trigger Type, both *F*s < 1. Further, in this case, the effect of Congruency Proportion was significant in a one-tailed *t*-test for both the task-irrelevant trigger (.267), *t*(71) = 1.90, *p* = .031, $\eta_{p}^{2}$ = .049, *BF*_+0_ = 1.38, and the task-relevant trigger (.367), *t*(71) = 2.47, *p* = .008, $\eta_{p}^{2}$ = .079, *BF*_+0_ = 4.36.

*Experiment 2*

Using IES, the main effects of Trigger Type (overall higher scores for stimuli involved in the task-relevant vs. task-irrelevant contrast), *F*(1,75) = 14.97, *MSE* = 5143, *p* < .001, $\eta_{p}^{2}$ = .166, and Congruency Proportion (higher scores for MC than MI stimuli overall), *F*(1,75) = 19.75, *MSE* = 8915, *p* < .001, $\eta_{p}^{2}$ = .208, were both significant. Their interaction was marginal, *F*(1,75) = 2.95, *MSE* = 8915, *p* = .090, $\eta_{p}^{2}$ = .038, reflecting the fact that the effect of Congruency Proportion tended to be smaller for the task-irrelevant trigger (39) than for the task-relevant trigger (56). Both of those effects, however, were significant when analyzed separately with a one-tailed *t*-test, *t*(75) = 2.95, *p* = .002, $\eta_{p}^{2}$ = .104, *BF*_+0_ = 13.35, and *t*(75) = 5.53, *p* < .001, $\eta_{p}^{2}$ = .290, *BF*_+0_ = 63466.56, respectively.

Using BIS, the only significant effect was that of Congruency Proportion (higher scores for MC than MI stimuli overall), *F*(1,75) = 25.61, *MSE* = .642, *p* < .001, $\eta_{p}^{2}$ = .255. There was neither a main effect of Trigger Type, *F*(1,75) = 2.57, *MSE* = 1.427, *p* = .113, $\eta_{p}^{2}$ = .033, nor an interaction between Congruency Proportion and Trigger Type, *F* < 1. Indeed, in this analysis, the Congruency Proportion effect had a similar size for the task-irrelevant trigger (.467) and the task-relevant trigger (.464), with both effects being significant when analyzed separately in one-tailed *t*-tests*, t*(75) = 3.37, *p* < .001, $\eta_{p}^{2}$ = .131, *BF*_+0_ = 41.67, and *t*(75) = 3.16, *p* = .001, $\eta_{p}^{2}$ = .118, *BF*_+0_ = 23.67, respectively.

References

Bruyer, R., & Brysbaert, M. (2011). Combining speed and accuracy in cognitive psychology: Is the inverse efficiency score (IES) a better dependent variable than the mean reaction time (RT) and the percentage of errors (PE)?. *Psychologica Belgica*, *51*, 5-13. https://doi.org/10.5334/pb-51-1-5

Crump, M. J., & Milliken, B. (2009). The flexibility of context-specific control: Evidence for context-driven generalization of item-specific control settings. *Quarterly Journal of Experimental Psychology*, *62*, 1523-1532. https://doi.org/10.1080/17470210902752096

Jacoby, L. L., Lindsay, D. S., & Hessels, S. (2003). Item-specific control of automatic processes: Stroop process dissociations. *Psychonomic Bulletin & Review*, *10*, 638-644. https://doi.org/10.3758/BF03196526

JASP Team (2020). *JASP* (Version 0.14.1) [Computer software].

Jeffreys, H. (1961). *Theory of probability* (3rd ed.). Oxford, UK: Oxford University Press.

Lee, M. D., & Wagenmakers, E. J. (2013). *Bayesian data analysis for cognitive science: A practical course*. New York, NY: Cambridge University Press.

Liesefeld, H. R., & Janczyk, M. (2019). Combining speed and accuracy to control for speed-accuracy trade-offs (?). *Behavior Research Methods*, 51, 40-60. https://doi.org/10.3758/s13428-018-1076-x

Spinelli, G., & Lupker, S. J. (2020). Item-specific control of attention in the Stroop task: Contingency learning is not the whole story in the item-specific proportion-congruent effect. *Memory & Cognition*, *48,* 426-435. https://doi.org/10.3758/s13421-019-00980-y

Townsend, J. T., & Ashby, F. G. (1983). *Stochastic modeling of elementary psychological processes*. Cambridge, UK: Cambridge University Press.
